# Country data on AMR in Türkiye in the context of community-acquired respiratory tract infections: links between antibiotic susceptibility, local and international antibiotic prescribing guidelines, access to medicine and clinical outcome

**DOI:** 10.1093/jac/dkac217

**Published:** 2022-09-06

**Authors:** Didem Torumkuney, Zerrin Aktas, Serhat Unal, James van Hasselt, Yalcin Seyhun, Nergis Keles

**Affiliations:** GlaxoSmithKline, 980 Great West Road, Brentford, Middlesex TW8 9GS, UK; Department of Clinical Microbiology, Istanbul Faculty of Medicine, Istanbul University, Istanbul, Türkiye; Department of Infectious Diseases and Clinical Microbiology, Hacettepe University Faculty of Medicine, Ankara, Türkiye; GlaxoSmithKline, The Campus, Flushing Meadows, 57 Sloane Street, Bryanston, Gauteng, 2021, South Africa; GlaxoSmithKline, Buyukdere Cad. No: 173, 1. Levent Plaza B Blok 34394 Levent, Istanbul, Türkiye; GlaxoSmithKline, Buyukdere Cad. No: 173, 1. Levent Plaza B Blok 34394 Levent, Istanbul, Türkiye

## Abstract

**Background:**

Antimicrobial resistance (AMR) is one of the biggest threats to global public health. Selection of resistant bacteria is driven by inappropriate use of antibiotics, amongst other factors. COVID-19 may have exacerbated AMR due to unnecessary antibiotic prescribing. Country-level knowledge is needed to understand options for action.

**Objectives:**

To review AMR in Türkiye and initiatives addressing it. Identifying any areas where more information is required will provide a call to action to minimize any further rise in AMR within Türkiye and to improve patient outcomes.

**Methods:**

National AMR initiatives, antibiotic use and prescribing, and availability of susceptibility data, particularly for the key community-acquired respiratory tract infection (CA-RTI) pathogens *Streptococcus pneumoniae* and *Haemophilus influenzae*, were identified. National and international antibiotic prescribing guidelines commonly used locally for specific CA-RTIs (community-acquired pneumonia, acute otitis media, acute bacterial rhinosinusitis) were also reviewed, plus local antibiotic availability. Insights from both a local clinician and local clinical microbiologist were sought to contextualize this information.

**Conclusions:**

Türkiye developed an antibiotic stewardship programme, The Rational Drug Use National Action Plan 2014–2017, prioritizing appropriate antibiotic prescription in the community. Public campaigns discouraging inappropriate antibiotic use were also initiated. Türkiye has a high level of antibiotic resistance and a high level of consumption, however, in 2015 over-the-counter antibiotic sales were prohibited, resulting in a declining trend in overall consumption. There is still a need for physician education on current developments in antibiotic use. Several ongoing global surveillance studies provide antibiotic susceptibility data in Türkiye. Clinicians in Türkiye use several country-specific guidelines for common CA-RTIs plus a range of international guidelines. A more standardized inclusive approach in developing local guidelines, using up-to-date surveillance data on isolates from community-acquired infections in Türkiye, could make guideline use more relevant for clinicians. This would pave the way for a higher level of appropriate antibiotic prescribing and improved adherence. This would, in turn, potentially limit AMR development and improve patient outcome.

## Introduction

Antimicrobial resistance (AMR) is one of the biggest threats to public health throughout the world^1^ as described in the introductory paper of this Supplement.^2^ The WHO states that ‘the world urgently needs to change the way it prescribes and uses antibiotics. Even if new medicines are developed, without behaviour change, antibiotic resistance will remain a major threat’.^3^ The first paper in this Supplement included details about the multiple factors which can drive a rise in AMR along with the global initiatives that are in place to address this threat.^2^ Each country and/or region must also play their part through local initiatives.

In order to identify how AMR can be addressed in Türkiye in the future, it is necessary to review what is happening now. In this paper, we present the current situation in Türkiye, determined using published information (from searching PubMed, Google Scholar and other internet platforms) to ascertain any national initiatives to address AMR in Türkiye, antibiotic use and prescribing, and availability of susceptibility data, in particular for the key community-acquired respiratory tract infection (CA-RTI) pathogens *Streptococcus pneumoniae* and *Haemophilus influenzae*. National and international antibiotic prescribing guidelines for CA-RTIs, specifically community-acquired pneumonia (CAP), acute otitis media (AOM) and acute bacterial rhinosinusitis (ABRS), commonly used by healthcare professionals in Türkiye were also reviewed, along with how these link to local antibiotic availability. Insights from a clinician and a clinical microbiologist in Türkiye were sought to contextualize this information. In addition, we aimed to identify areas where more information is required and present a call to action to improve clinical outcome for patients and to minimize further rises in AMR within Türkiye.

## Action Plans

Following the formulation by the World Health Assembly in 2015 of a Global Action Plan (GAP) for AMR^4^ many countries began to develop their own National Action Plan (NAP). To address the problem of antibiotic overconsumption and resistance, Turkish health authorities implemented several integrated interventions with WHO guidance and support. Türkiye developed an antibiotic stewardship programme, The Rational Drug Use National Action Plan 2014–2017, which prioritized the proper use of antibiotics in the community. Public campaigns discouraging inappropriate antibiotic use were initiated, and, in 2015, over-the-counter sales of antibiotics were prohibited, resulting in a declining trend in overall consumption with the proportion of antibiotics prescribed by family practitioners dropping by 25% in 2017.^5^ Current NAP status, as reported by the WHO for 2020–21, shows that Türkiye had yet to finalize an AMR NAP.^6^

## Antibiotic use and AMR

The Organisation for Economic Co-operation and Development reported in 2015 that, amongst its member countries, Türkiye had one of the highest rates of antibiotic resistance at seven times higher than the lowest rates seen.^5^ Resistance development is frequently associated with inappropriate antibiotic use.^7^ According to the WHO, the consumption of antibiotics in in Türkiye was 38.2 DDD per 1000 inhabitants per day in 2015. This was the highest consumption reported in the WHO European region comprising 45 countries.^8^

A retrospective year-long descriptive study in 2017 throughout Türkiye of e-prescription data from family physicians showed that around one-quarter of all prescriptions included at least one antibiotic.^9^ In another study surveying public knowledge about antibiotic use, in 1044 participants in Ankara, about 24% of participants had demanded antibiotics when visiting a physician and 83.5% were successful in their request, indicating that more physician education is needed.^10^

In 2018, the WHO Regional Office for Europe Antimicrobial Medicines Consumption (AMC) Network, revealed that for Türkiye there was an ongoing reduction in antibiotic consumption estimates showing a continued national effort to improve antimicrobial use.^11^ This was further demonstrated by an 8 year evaluation of more than a billion outpatient prescriptions (from general practitioners) in all age groups. At the start of the study, in 2011, 34.9% of the prescriptions contained at least one antibiotic but this fell to 24.6% in 2018. In 2011, 14% of all items prescribed were antibiotics, but this reduced to 10.5% in 2018 and during the same period, the percentage of total drug costs accounted for by antibiotics fell from 14.1% to 4.1%. The study concluded that the national interventions had been effective in reducing antibiotic use (and at the same had raised the preference of first-line antibiotics at primary healthcare level in Türkiye over a course of 8 years).^12^

A study investigating knowledge and awareness of physicians concerning rational antibiotic use in a Turkish province revealed that differences in knowledge, attitudes, and behaviours of primary care physicians on the decision to start treatment and its necessity may account, at least in part, for increased rates; and that pressure from patients and their relatives may encourage prescribing. Awareness of multidrug resistance is low. The fear of unproven infection seems to be the most important factor that encourages doctors to start antibiotics. There is still a need for ongoing education for physicians on current developments in antibiotic use.^13^

A new Turkish version of Antibiotic Guardian, a web-based campaign initiated by Public Health England in 2014, has been made available. Antibiotic Guardian urges everyone to make a pledge to make better use of antibiotics and help save antibiotics from becoming obsolete. To date more than 50^ ^000 people in Türkiye have pledged their support for the campaign.^14^

## Surveillance

### Global surveillance studies

#### SOAR

Several ongoing global surveillance studies provide antibiotic susceptibility data in Türkiye. The Survey of Antibiotic Resistance (SOAR) is a multinational antibiotic surveillance study, ongoing in an expanding range of countries since 2002. The study aims to collect and make available in published, peer-reviewed papers, antibiotic susceptibility data, specifically for *S. pneumoniae* and *H. influenzae*, the most commonly isolated respiratory pathogens in the community.^15^ Key features of SOAR are that it focusses on these pathogens only, and that identification and susceptibility testing are performed in an independent centralized laboratory using standardized methodology (CLSI). SOAR data is analysed based on three different breakpoints: CLSI, EUCAST dose-specific and PK/PD breakpoints. This allows for comparisons to be made between countries or regions and for the identification of trends over time.

Clinical breakpoints are cut-off MIC values used to classify microorganisms into the clinical categories susceptible (S), intermediate (I) and resistant (R) in order to assist in predicting the clinical success or failure of a specific antibiotic.^16^ Two international organizations define breakpoint values: CLSI and EUCAST. Due to variation in criteria for their definition, there are some differences between CLSI and EUCAST in the clinical breakpoint values for certain bacteria for some antibiotics and this can impact susceptibility interpretation of clinical isolates.^17^ EUCAST breakpoints are dose-specific and use the EMA-approved doses that are included in the Summary of Product Characteristics of an antibiotic. This means that by application of breakpoints for higher doses, the effect of using a raised dose on the clinical efficacy of a particular antibiotic can be predicted. Türkiye is in a transition period from CLSI to EUCAST guidelines and breakpoints.^18^ It is therefore possible that dose-specific breakpoints could be used commonly in Türkiye in future. The EUCAST dose-specific breakpoints can also be used retrospectively to calculate the susceptibility of previously collected isolates to show the susceptibility levels that would have been achieved at higher doses.

Use of the EUCAST dose-specific breakpoints shows the effect of increasing the antibiotic dose on the susceptibility of a pathogen, providing additional information so the prescriber can decide if a higher dose would be of benefit. For example, *S. pneumoniae* (the most commonly isolated respiratory pathogen^19,20^ for clinical conditions such as CAP, AOM and ABRS) has over time become less susceptible to amoxicillin/clavulanic acid in some countries^21^ since the MICs for some isolates have increased. When treating infections, it is important to be able to eradicate bacterial pathogens with raised MICs to optimize clinical outcome while at the same time minimizing the risk of selecting variants with even higher MICs. This is possible because β-lactams, unlike many other antibiotics, have time-dependent killing properties. Their efficacy depends on the amount of time the drug concentration is present at the site of action. Although increasing the concentration at the infection site over a particular concentration will not have any effect on the efficacy, the use of higher doses and/or more frequent dosing allows for successful eradication of pathogens with higher MICs because the time above the MIC is increased.

**Figure 1.**
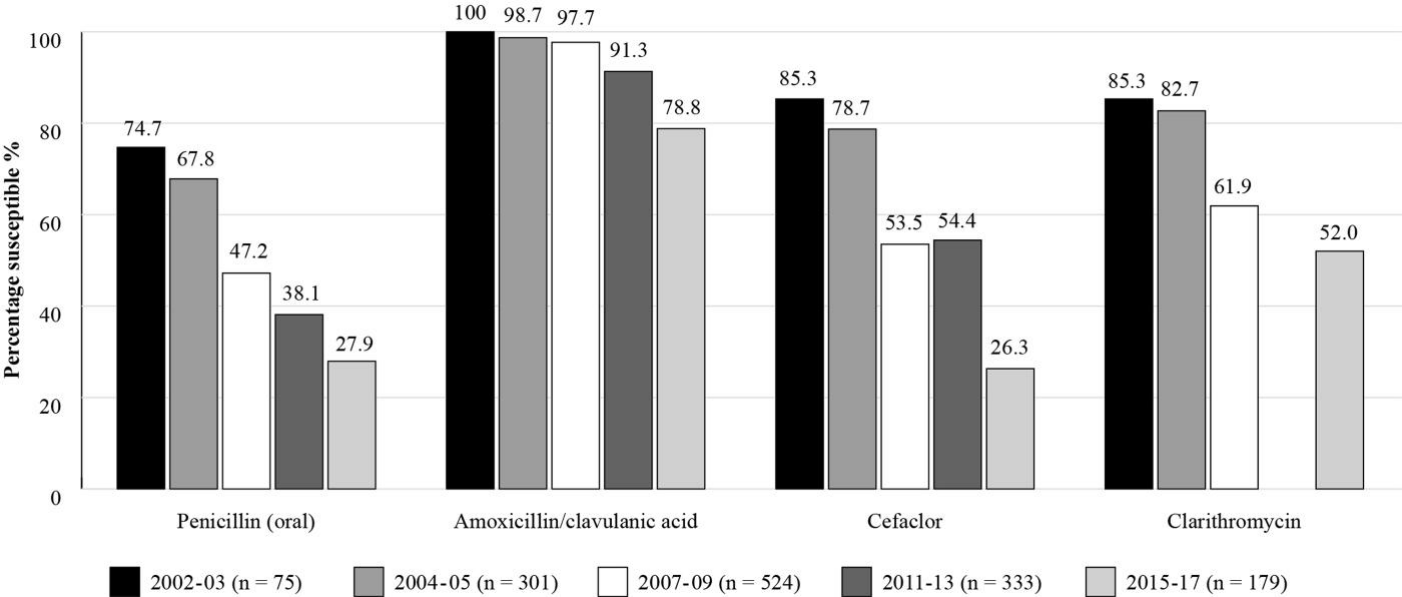
Percentage susceptibility rates based on CLSI breakpoints for antibiotics against all *S. pneumoniae* isolates collected as part of the SOAR study in Türkiye for five time periods.

Türkiye has participated in the SOAR studies over five time periods, starting in 2002 and ending in 2017, and comparative results, as shown in Figure 1, clearly show the steady downward trend in susceptibility of *S. pneumoniae* isolates for all the antibiotics tested.^15,21,22^

**Figure 2.**
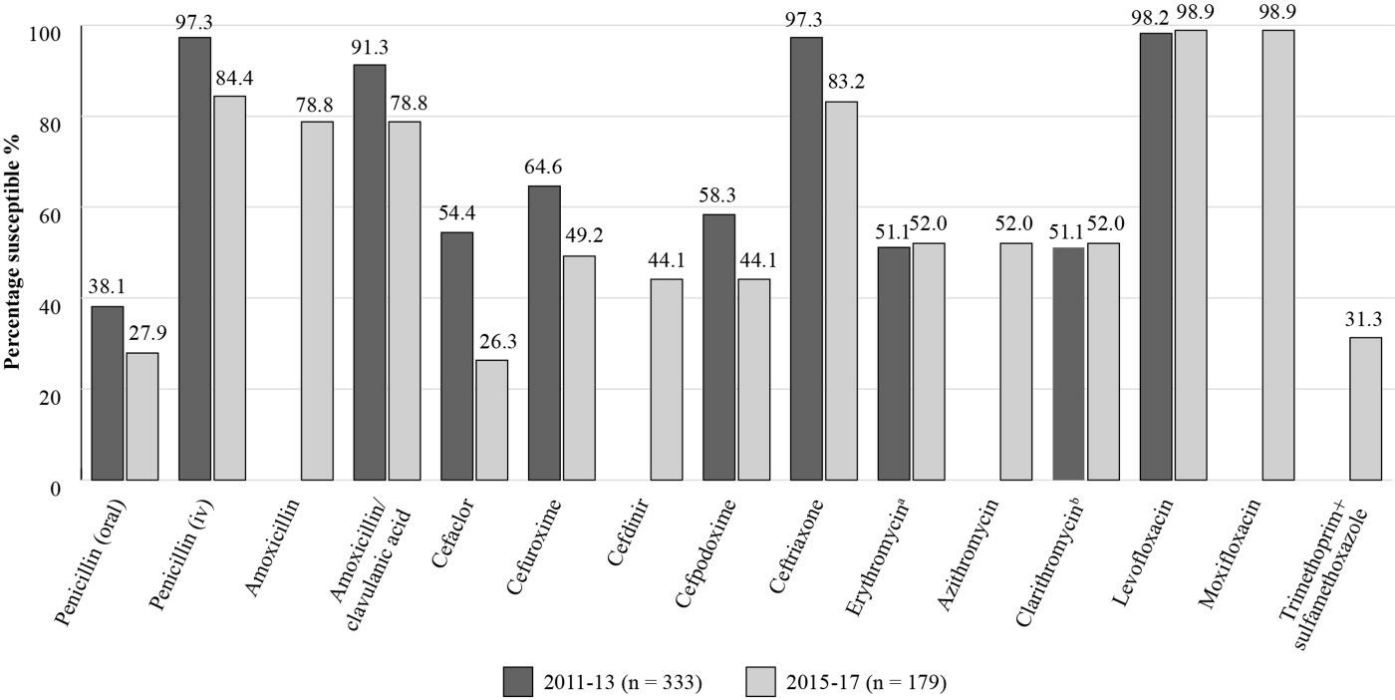
Percentage susceptibility rates based on CLSI breakpoints for antibiotics against all *S. pneumoniae* isolates collected as part of the SOAR study in Türkiye in 2011–13 and 2015–17. ^a^321 isolates tested in 2011–13. ^b^2011–13 based on percentage susceptibility to erythromycin, CLSI guidelines.

Figure 2 shows the SOAR results for *S. pneumoniae* isolates in Türkiye for the two most recent time periods studied.^15,22^ In 2015 to 2017, 179 *S. pneumoniae* isolates from outpatients with CA-RTIs were collected from three centres. When applying CLSI breakpoints 78.8% of isolates were susceptible to amoxicillin and amoxicillin/clavulanic acid in 2015–17, with susceptibility of 52% to erythromycin, azithromycin, clarithromycin and between 26.3%–49.2% susceptibility to cefaclor, cefdinir, cefpodoxime, and cefuroxime. Prevalence of susceptibility to ceftriaxone was 83.2%.^15^

**Figure 3.**
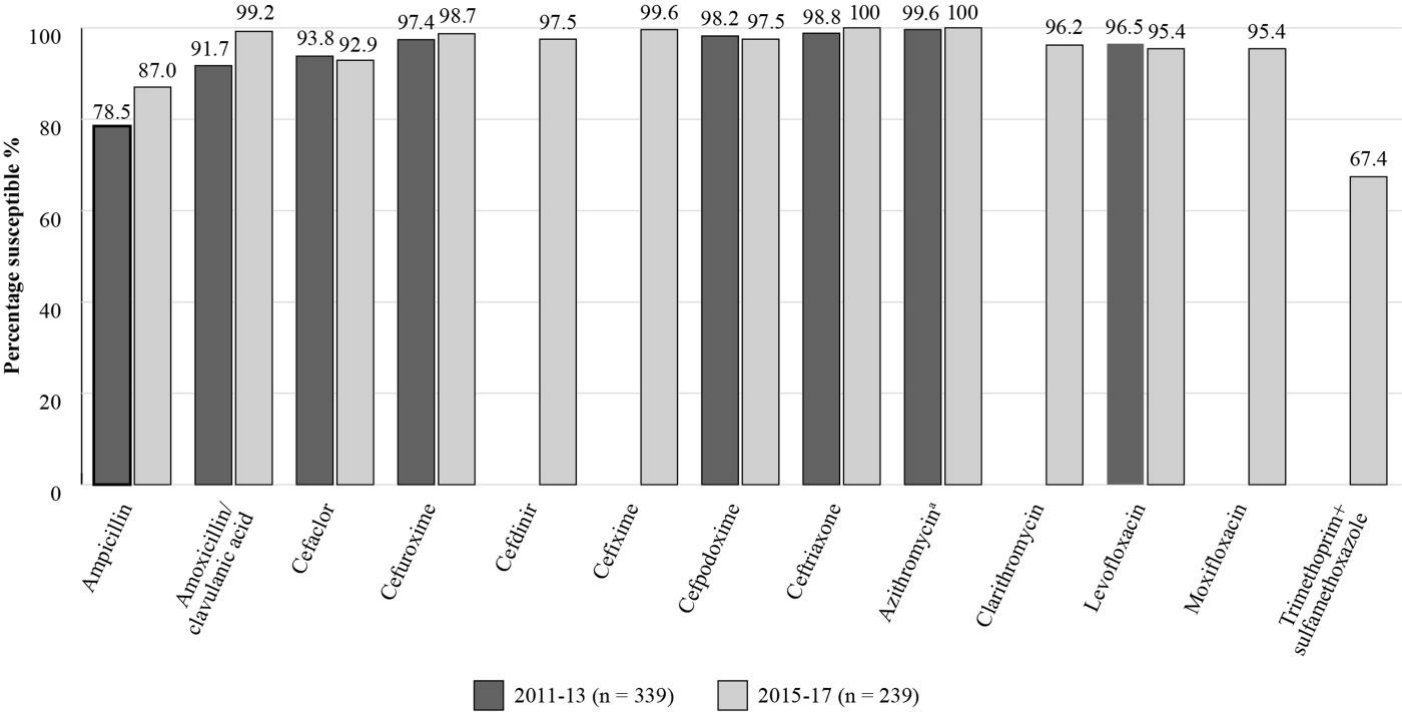
Percentage susceptibility rates based on CLSI breakpoints for antibiotics against all *H. influenzae* isolates collected as part of the SOAR study in Türkiye in 2011–13 and 2015–17. ^a^230 isolates tested in 2011–13.

Figure 3 shows a plot of the SOAR results for *H. influenzae* isolates in Türkiye for the two most recent time periods studied.^15,22^ For *H. influenzae* isolates (*n *= 239), in the 2015–17 study, a high susceptibility to amoxicillin/clavulanic acid was seen (99.2%) along with all other antibiotics tested (except trimethoprim/sulfamethoxazole for which susceptibility was 67.4%) when applying CLSI breakpoints. Most isolates of *H. influenzae* from Türkiye were β-lactamase negative in 2015–17, but the presence of some β-lactamase-positive isolates meant that the susceptibility of *H. influenzae* to ampicillin was lower.^15^

#### ATLAS

The Antimicrobial Testing Leadership and Surveillance (ATLAS) database is a global AMR surveillance programme that is fully accessible and covers susceptibilities of a range of bacterial and fungal pathogens to a bank of antimicrobials with reference to the different breakpoints.^23^ ATLAS data is analysed based on CLSI and EUCAST breakpoints.

**Figure 4.**
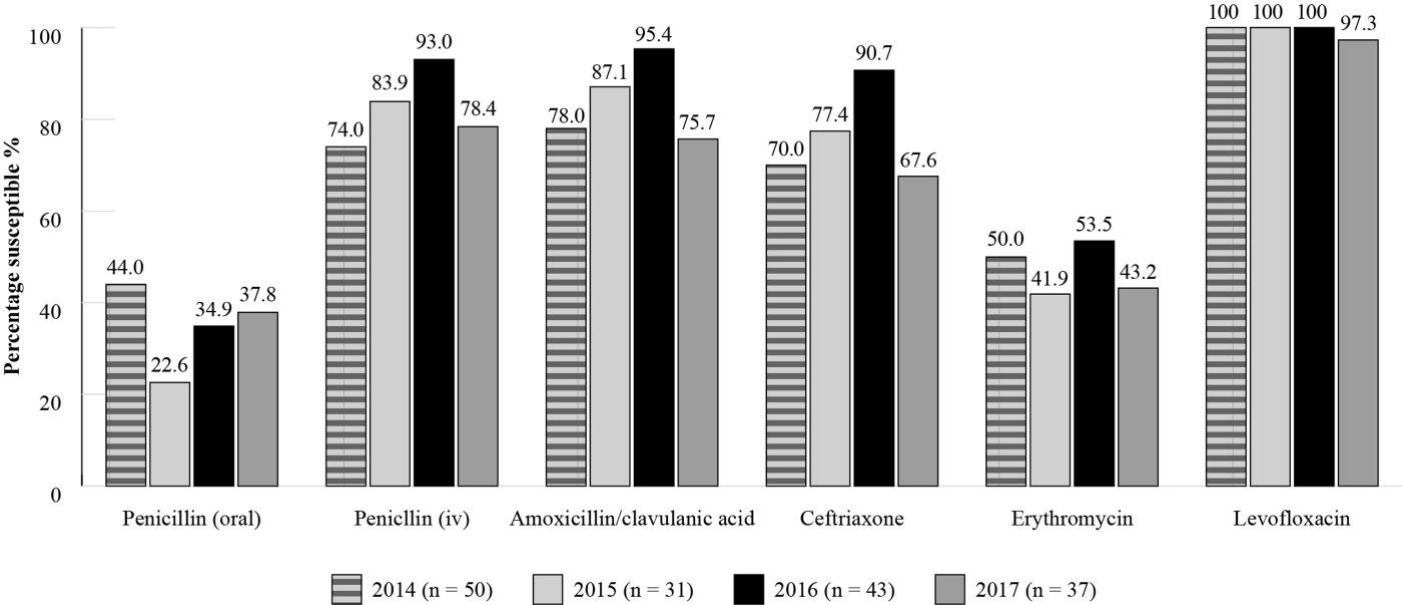
Percentage susceptibility rates based on CLSI breakpoints for antibiotics against *S. pneumoniae* isolates collected as part of the ATLAS study in Türkiye in 2014–17. Data access date 22 November 2021.

**Figure 5.**
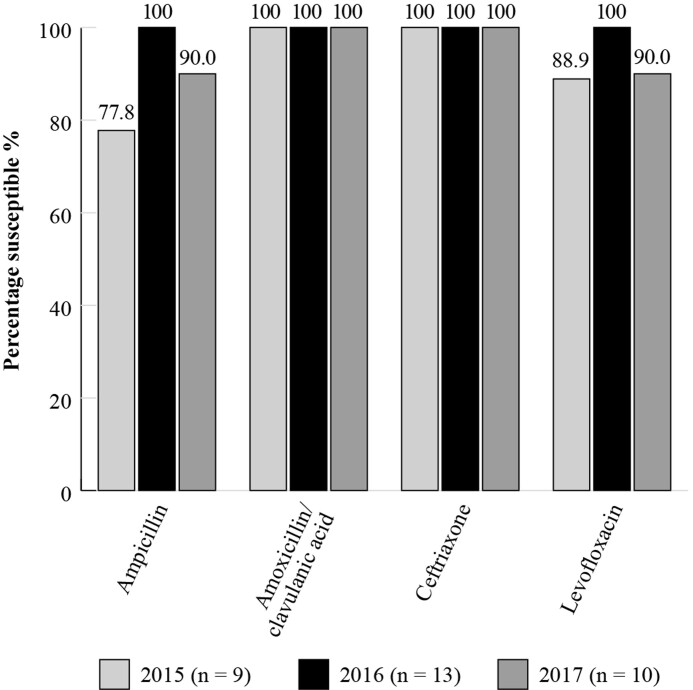
Percentage susceptibility rates based on CLSI breakpoints for antibiotics against *H. influenzae* isolates collected as part of the ATLAS study in Türkiye in 2015–17. Data access date 22 November 2021.

Susceptibility data is available for *S. pneumoniae* isolates collected between 2014 and 2017 (Figure 4: 2014 *n *= 50; 2015 *n *= 31; 2016 *n *= 43; 2017 *n *= 37) and for *H. influenzae* isolates between 2015 and 2017 (Figure 5: 2015 *n *= 9; 2016 *n *= 13; 2017 *n *= 10). Using CLSI criteria, susceptibility of *S. pneumoniae* isolates to amoxicillin/clavulanic acid was between 75.7% and 95.4% over this period. This contrasts with 41.9%–53.5% for erythromycin, where erythromycin susceptibility also represents that for other macrolides such as azithromycin and clarithromycin based on CLSI guidelines (Figure 4). Confirming the results shown in the high susceptibility values seen in the most recent SOAR study, the ATLAS study showed that *H. influenzae* isolates remained fully susceptible to amoxicillin/clavulanic acid and ceftriaxone in 2017, applying CLSI breakpoints (Figure 5).^23^

#### SENTRY

**Figure 6.**
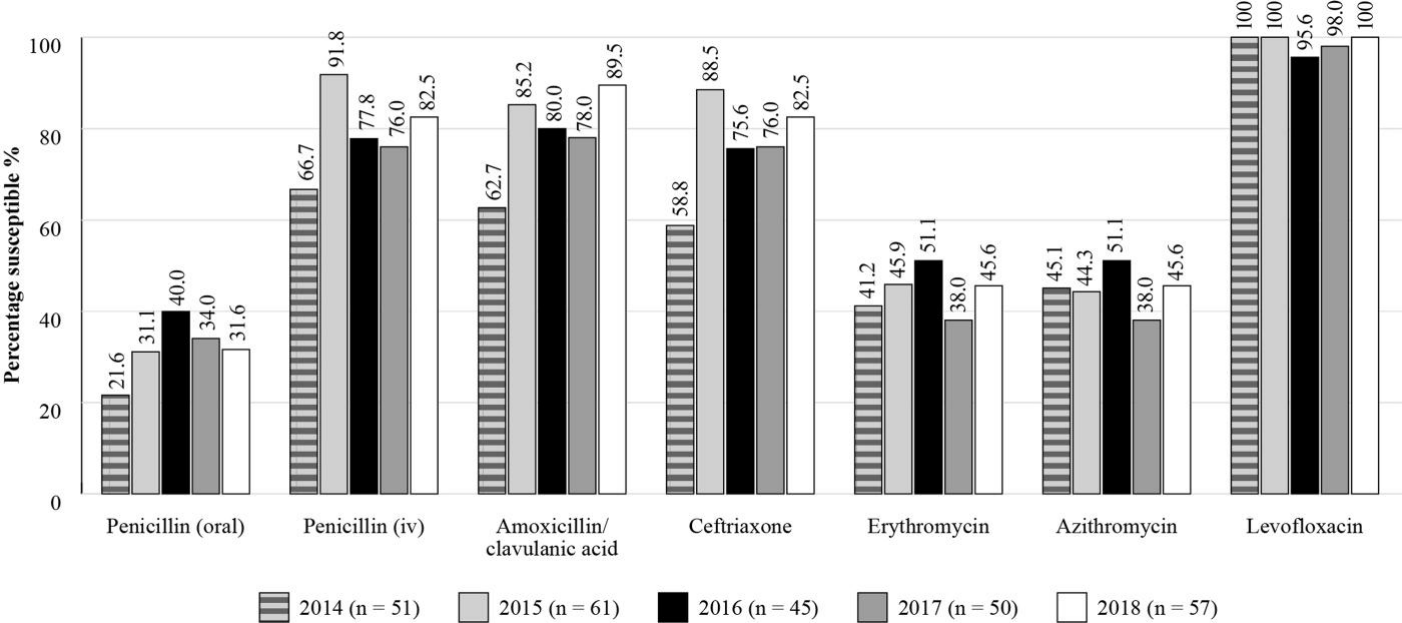
Percentage susceptibility rates based on CLSI breakpoints for antibiotics against *S. pneumoniae* isolates collected as part of the SENTRY study in Türkiye in 2014–18. Data access date 22 November 2021.

The SENTRY Antimicrobial Surveillance Program was established in 1997 and is another long-running antimicrobial surveillance programme. SENTRY monitors worldwide pathogens and changes in resistance patterns over time through centralized testing and utilizing reference susceptibility methods.^24^ Between 2014 and 2018, the susceptibility of *S. pneumoniae* respiratory isolates (Figure 6: 2014 *n *= 51; 2015 *n *= 61; 2016 *n *= 45; 2017 *n *= 50; 2018 *n *= 57) to amoxicillin/clavulanic acid, intravenous penicillin and ceftriaxone were reasonably consistent and high over the last 4 years tested, whilst the macrolides (erythromycin and azithromycin) and penicillin (oral) showed a much lower level of susceptibility over the same time period. Susceptibility to the fluoroquinolone antibiotics remained high throughout the period under investigation. The results for the susceptibility of *H. influenzae* isolates confirmed that the respiratory isolates collected were highly susceptible to almost all the antibiotics tested, including amoxicillin/clavulanic acid.

#### GLASS

In 2015, the WHO launched the Global Antimicrobial Resistance and Use Surveillance System (GLASS). GLASS is the first global system to collect national AMR data for selected bacterial pathogens that cause common infections. The aim is to monitor the prevalence of AMR among major pathogens in clinical settings^25^ to provide the supporting data required to ensure that countries can design cost-effective, evidence-based AMR response strategies. During the first four years, 91 countries or territories enrolled in GLASS, and data for over two million patients from 66 countries are included.^26^ Pathogens currently included in GLASS-AMR are: *Acinetobacter* spp., *Escherichia coli*, *Klebsiella pneumoniae*, *Neisseria gonorrhoeae*, *Salmonella* spp., *Shigella* spp., *Staphylococcus aureus*, and *S. pneumoniae.* GLASS data is analysed based on CLSI and EUCAST breakpoints. A new and important component is the inclusion of antimicrobial consumption (AMC) surveillance at the national level.^27^ Türkiye is currently not participating in GLASS.

#### CAESAR

Türkiye is a member of the Central Asian and Eastern European Surveillance on Antimicrobial Resistance (CAESAR) network, a joint initiative of the WHO/Europe and partners to survey, contain and prevent AMR emergence and spread. Türkiye has submitted some data, but isolates collected are mainly from hospitals and invasive pathogens. The *S. pneumoniae* isolates included, for example, are from blood and cerebrospinal fluid.^28^

## Disease Management Guidelines

For management of the common RTIs, CAP, AOM and ABRS in Türkiye, clinicians make use of several country-specific local antibiotic prescribing guidelines plus a range of international guidelines, some examples of which are included in Table 1. Most guidelines suggest a first-line antibiotic or antibiotics along with alternative(s) and then a second-line antibiotic or antibiotics, also with an alternative(s). The first-line antibiotic is the recommended first choice that should be prescribed by the clinician following diagnosis of the infection, supported by criteria defined by the organization or committee; alternative(s) may be provided for use in particular circumstances. For example, if the first-line antibiotic is a β-lactam then alternative suggestions will be for use in the case of penicillin allergy. The second-line antibiotic is for use if the first-line does not achieve the anticipated outcome, and again alternative(s) may be included for use under specific circumstances.

**Table 1.** Examples of the main local and international antibiotic prescribing guidelines referred to by physicians in Türkiye for the management of community-acquired respiratory tract infections

Local antibiotic prescribing guidelines
Turkish Thoracic Society 2009: Diagnosis and Treatment of Community-acquired Pneumonia in Children^29^
Turkish Thoracic Society 2021: Diagnosis and Treatment of Community-acquired Pneumonia in Adults^30^
International antibiotic prescribing guidelines
BTS 2009: British Thoracic Society Guidelines for the Management of Community-acquired Pneumonia in Adults: update 2009^31^
IDSA 2011 (Endorsed by AAP): The Management of Community-Acquired Pneumonia in Infants and Children Older Than 3 Months of Age: Clinical Practice Guidelines by the Pediatric Infectious Diseases Society and the Infectious Diseases Society of America^32^
BTS 2011: British Thoracic Society guidelines for the management of community-acquired pneumonia in children: update 2011^33^
IDSA 2012: IDSA Clinical Practice Guideline for Acute Bacterial Rhinosinusitis in Children and Adults^34^
AAP 2013: American Academy of Pediatrics. The diagnosis and management of acute otitis media^35^
AAP 2013: American Academy of Pediatrics: Clinical practice guideline for the diagnosis and management of acute bacterial sinusitis in children aged 1 to 18 years Clinical Practice Guideline for the Diagnosis and Management of Acute Bacterial Sinusitis in Children Aged 1 to 18 Years^36^
American Academy of Family Physicians 2013: Otitis media: diagnosis and treatment^37^
American Academy of Otolaryngology-Head and Neck Surgery Foundation Guideline 2015: Clinical Practice Guideline (Update): Adult Sinusitis^38^
NICE 2017: Sinusitis (Acute): Antimicrobial Prescribing^39^
IDSA 2019: Diagnosis and Treatment of Adults with Community-acquired Pneumonia. An Official Clinical Practice Guideline of the American Thoracic Society and Infectious Diseases Society of America^40^
WHO 2019: World Health Organization Model List of Essential Medicines^41^

### International antibiotic prescribing guidelines

For the management of CAP, the guidelines principally followed by physicians in Türkiye are the IDSA 2019 guidelines for adults^40^ and for children the Paediatric Society of America/IDSA 2011 guidelines.^32^ The British Thoracic Society (BTS) guidelines for adults 2009,^31^ and for children the IDSA 2011 guidelines are used^33^ as well as the WHO guidelines, as provided in the List of Essential Medicines.^41^ For example, a first-line antibiotic treatment recommendation for CAP management from the IDSA 2019 guideline for treating adults with no comorbidities or risk factors for MRSA or *Pseudomonas aeruginosa*, is amoxicillin or doxycycline or a macrolide (if the local pneumococcal resistance is <25%) but if the patient has comorbidities, the recommendation is combination therapy with amoxicillin/clavulanic acid or a cephalosporin plus a macrolide or doxycycline, or monotherapy with a respiratory fluoroquinolone. The recommended dosage for adults with comorbidities is amoxicillin/clavulanic acid, 500 mg/125 mg given three times daily, 875 mg/125 mg or 2000 mg/125 mg both given twice daily in combination with a macrolide or doxycycline.^40^ The BTS also suggests amoxicillin/clavulanic acid, cefaclor, and macrolides as an alternative to first-line amoxicillin in children with low-severity pneumonia.^33^

In AOM, the American Academy of Paediatrics (AAP) guidelines are followed which specifically recommend high dose amoxicillin (80–90 mg/kg/day) given in two divided doses or amoxicillin/clavulanic acid 14:1 formulation, 90 mg/kg/day amoxicillin with 6.4 mg clavulanic acid, given in two divided doses for first-line treatment in children with initial or delayed antibiotic treatment and also recommends the high dose amoxicillin/clavulanic acid 14:1 formulation, 90 mg/kg/day amoxicillin with 6.4 mg clavulanic acid, given in two divided doses or ceftriaxone for patients who have failed initial antibiotic treatment.^35^

For the management of ABRS in adults and children, guidelines used include those from the IDSA.^34^ According to the IDSA guideline, amoxicillin/clavulanic acid 90 mg/kg/day given twice daily is recommended as an empirical therapy for children in certain circumstances such as severe disease or in areas where penicillin non-susceptible *S. pneumoniae* are endemic.

### National antibiotic prescribing guidelines

National guidelines in line with more recent international guidelines were published by the Turkish Thoracic Society in 2009 for CAP in children and in 2021 for CAP in adults as contributed by many local associations.

## Antibiotic availability

Access to antibiotics may be an issue for patients in low- and middle-income countries (LMICs) due to cost and insufficient government expenditure or support in this area. Unreliable drug supply may also contribute to the problem. Limited access to the most appropriate antibiotic to treat a specific infection may result in raised mortality from treatable bacterial infections. The use of suboptimal amounts of antibiotic facilitates resistance development and allows resistant strains to persist.^41–43^

In Türkiye, using amoxicillin/clavulanic acid as an example, several formulations of this antibiotic are currently available, however, the dosage and indication for all forms of amoxicillin/clavulanic acid are currently under review by the Turkish Ministry of Health (MoH). The formulations of amoxicillin/clavulanic acid available in Türkiye are all given twice daily. In outpatients with CAP and comorbidities the IDSA 2019 guidelines^40^ recommend for adults, amoxicillin/clavulanic acid 875 mg/125 mg twice daily (or 2000 mg/125 mg twice daily) as an alternative to the three times daily 500 mg/125 mg regimen. In children who are outpatients with bacterial pneumonia and comorbidities, a dose of 90 mg/kg/day in two divided doses (as amoxicillin component) is suggested by the IDSA.^32^

In ABRS, the IDSA guidelines^34^ recommend as standard first-line therapy amoxicillin/clavulanic acid 500 mg/125 mg three times daily or 875 mg/125 mg twice daily in adults and 45 mg/kg/day twice daily in children. ‘High dose’ regimens (2 g given twice daily in adults and in children 90 mg/kg/day given twice daily) are recommended as a second-line empirical therapy or in situations such as severe disease or where there is endemic penicillin non-susceptible *S. pneumoniae*. In AOM, the AAP^35^ recommend amoxicillin/clavulanic acid 90 mg/kg/day (as amoxicillin component) in two divided doses (formulation 14:1) as first-line for children for initial or delayed antibiotic treatment or after failure of initial antibiotic treatment.

Substandard poor-quality or falsified antibiotics promote AMR and the spread of drug-resistant infections. Since poor-quality antibiotics are unlikely to contain the full dose needed to eliminate all of the infecting pathogens, use of these would encourage resistance to develop and allow resistant strains to survive and be transmitted.^44^ The quality of medicines, specifically antibiotics, is an important consideration for countries worldwide. WHO has launched a Global Surveillance and Monitoring System (GSMS) for substandard and falsified products.^44^ The GSMS aims to work with WHO member states to improve the quality of reporting of substandard and falsified medical products, and, importantly, to ensure the data collected are analysed and used to influence policy, procedure, and processes to protect public health, at the national, regional and the global level. Use of substandard or falsified antibiotics not only compromises clinical outcomes but also risks increased AMR. The most recent summary (2013–17) reported substandard and falsified medicines in 46 member states (including Türkiye). Antibiotics represent 16.9% of all products reported, second only to malaria drugs (19.6%).

## Local insights

### Clinical microbiologist expert comment

Surveillance studies provide important information allowing the identification of trends in pathogen incidence and AMR, including identification of emerging pathogens at local hospital, regional, national and global levels. Local surveillance studies indicate that the incidence of AMR pathogens is increasing and can guide clinician decisions regarding appropriate treatment and to modify treatment guidelines and aid in empirical prescribing. Microbiological methods used for AMR detection in hospitals in Türkiye generally conform to EUCAST and CLSI antibiotic susceptibility testing. Local surveillance studies are also a large part of efforts to assess AMR patterns and provide important information to guide prescribing at individual facilities. At the same time, surveillance studies need to maintain a mix of the small and the large, the local and the global. The Turkish MoH is the main regulator and provider of healthcare in Türkiye. In the absence of standardized national surveillance, some universities generate annual reports of their AST results.^5^ There is a need for a robust national AMR surveillance system in Türkiye, which could be included in the already present global systems for providing accurate and reliable surveillance data. Türkiye has two main antimicrobial stewardship programmes established by the MoH, targeting hospitals and the community.

Rapid delivery of microbiology test results influences mortality, length of hospital stay and costs, as well as appropriateness of antibiotic prescribing and consumption. In Türkiye, Electronic Health Records include any information recorded, stored, transmitted, accessed, correlated, and processed by using electronic systems relating to physical/mental health conditions or diseases of individuals.^23^ Many hospitals in Türkiye have electronic medical record systems and most have integrated Laboratory Information Systems. Rarely, vital test results may not be received by the treating clinicians, posing a patient safety threat and reflecting a need for common protocols on who is to receive test results and how to better integrate laboratory IT systems and clinical units. In addition to traditional disease notification systems, it is important to switch to real-time event management, which would significantly reduce turnaround time. Close monitoring of hospital antibiotic use for restricted and unrestricted antibiotics is essential. Resistance rates for key community pathogens should be monitored and antibiotic surveillance needs to be extended to outpatient clinics.

New rapid microbiological techniques should replace traditional culturing and susceptibility testing. In addition, revision of testing processes may improve workload, and workflow. Another solution to enhance quality control could be artificial intelligence-based systems. The microbiologists could also supervise clinicians via tele-microbiology service when interpreting Gram stains and results of culture. Providing the right treatment within the first 24–48 h of symptoms is essential.

Educational programmes combined with mutual internships for laboratory and clinical staff could contribute to a better understanding of complementary work processes. In performing antibiotic stewardship outreach visits in clinical units, microbiologists can enhance their role as interpreters of clinical processes and patient information to the laboratory technicians. Such visits also provide an opportunity to convey microbiology insights to clinical staff and teach them how to better interpret test results. In future, microbiological methods will evolve and become increasingly sophisticated and the need for professional guidance will increase. This may require a change in how some microbiologists execute their profession, from being predominantly laboratory based to working more closely with clinical staff. It is desirable to provide laboratory evidence of diseases not just at the level of clinical diagnosis, so it is necessary to develop both human and diagnostic capacities of laboratories.

### Clinician expert comment

Türkiye has very high antibiotic consumption and RTIs are among the diseases for which antibiotics are most frequently used. Administration of treatment in accordance with guideline recommendations not only ensures correct, appropriate, and necessary treatment, but may also reduce the development of AMR. Guideline recommendations, empirical treatment principles and local surveillance data should be considered before prescribing antibiotics. Considering the evaluation and publication times of local surveillance data, shorter intervals and faster and easier access to up-to-date data may be more beneficial for the application of ‘patient-specific treatments’. This ‘patient-focused approach’ should be evaluated together with the guideline recommendations as well as the clinical features of patients, disease characteristics, risks of complication and pharmacokinetic/pharmacodynamic (PK/PD) characteristics of the preferred antibiotic(s).

The results of local surveillance studies conducted in different cities or clinics in Türkiye have been published but they do not show continuity. As described here, SOAR Türkiye studies have been published for five different time periods and continuous participation of three centres is valuable for monitoring national and regional results and changes.^15,21,22^

In Türkiye, there are no local guidelines on the treatment management of diseases such as sinusitis, otitis, tonsillopharyngitis in children and adults; but the booklet entitled ‘Rational Approach to Antibiotic Use in Adult Patients’ published by the MoH, Turkish Medicines and Medical Devices Agency is defined as a ‘local guideline’ that indicates the appropriate and rational use of antibiotics in the treatment of many diseases, including respiratory tract infections.^45^ Recommendations of international guidelines should be considered along with local guidelines when prescribing appropriate antibiotics specific to the disease and the patient. In addition, bacteria-specific local antibiotic resistance data together with endemic resistance rates (especially for *S. pneumoniae*) should be considered.

When prescribing antibiotics in Türkiye, the ‘Electronic Prescription System’ program is used in which all antibiotics licensed in Türkiye are listed. Physicians can choose the appropriate antibiotic for their patient with recommended dose and treatment duration. The licensed antibiotics in Türkiye provide sufficient variety for the treatment of many diseases so problems of treatment choice are minimal, especially for adults. Most antibiotic consumption is from primary care and acute RTIs are the most common reason for prescribing antibiotics, particularly in primary care, where antibiotics are often prescribed inappropriately. Ensuring the rational use of antibiotics is a priority within the scope of the National Action Plan for Rational Use of Medicines 2014–2017, which was put into practice in 2014 in order to contribute to slowing AMR.

## Conclusions

In an era of rising AMR throughout the world, this paper aims to define areas where action is required to address AMR by analysing and understanding the current situation within a country or region. Information is presented for Türkiye concerning antibiotic use and prescribing, approach to AMR, availability of local susceptibility data, use of international and/or local management guidelines and how these link to antibiotic availability. To our knowledge this is the first time this information has been reviewed and presented in detail by country.

Antibiotic use in Türkiye is extremely high. This and the lack of knowledge about antibiotic susceptibilities has led to a high degree of antibiotic resistance. Türkiye has the highest consumption of antibiotics per capita in the WHO European region. There is still a need for ongoing education for physicians on current developments in antibiotic use.

In terms of surveillance, Türkiye is a member of the CAESAR network, a joint initiative of WHO/Europe.^28^ Several global surveillance studies have collected and published data from Türkiye for the principal pathogens in CA-RTIs including SOAR, ATLAS and SENTRY. Surveillance study results consistently show a decrease in susceptibility of respiratory pathogens for several classes of antibiotics, in particular the macrolides. Susceptibility to the fluoroquinolone antibiotics remains high in Türkiye amongst respiratory pathogens but regulatory bodies advise caution for their use, recommending restriction to specific cases, due to safety concerns.

Initiatives to address the overconsumption of antibiotics and antibiotic resistance in Türkiye have had success, for example, in reducing the antibiotic prescriptions by family practitioners by 25%. However, further improvements, monitoring and investment are required to sustain Türkiye’s antibiotic stewardship programmes.

Whilst a range of guidelines are utilized by clinicians in Türkiye, a more standardized inclusive approach is needed to develop local country-specific guidelines These guidelines should be based on up-to-date surveillance data of isolates from community-acquired infections which would make them more locally relevant for clinicians, reiterating the Consensus Principles as described in the introductory paper to this Supplement. This would pave the way for improved adherence and a higher level of appropriate antibiotic prescribing in CA-RTIs which could, in turn, potentially limit AMR development and improve clinical outcomes for patients.
